# Ultra-Low Tidal Volumes And Extracorporeal Carbon Dioxide Removal (Hemolung^®^ Ras) in Ards Patients. a Clinical Feasibility Study

**DOI:** 10.1186/2197-425X-3-S1-A7

**Published:** 2015-10-01

**Authors:** FJ Parrilla, L Bergesio, H Aguirre-Bermeo, JC Suarez, P López, I Morán, J Mancebo

**Affiliations:** Hospital de la Santa Creu i Sant Pau, Medicina Intensiva, Barcelona, Spain

## Introduction

Ventilation of ARDS patients with low tidal volume (Vt) is performed in order to minimize ventilation induced lung injury. This strategy, however, may induce hypercapnic acidosis, promote derecruitment and, in some individuals, induce alveolar overdistention despite the use of low Vt. Extracorporeal CO_2_ removal can help minimizing hypercapnic acidosis and to further reduce Vt (i.e. *ultraprotective* ventilation).

## Objectives

To evaluate the effect of extracorporeal CO_2_ removal in ARDS during *ultraprotective* ventilation in terms of lung mechanics and gas exchange.

## Methods

We studied 9 ARDS patients, in whom *ultraprotective* ventilation (i.e. Vt 4 ml/kg PBW) was implemented by means of an extracorporeal CO_2_ removal system [Hemolung^®^ Respiratory Assist System (RAS), ALung, Pittsburgh]. Anticoagulation with unfractionated heparin to reach an aPTT target range of 1.5-2 was used. We compared baseline ventilation with *ultraprotective* ventilation (combining Vt of 4 ml/kg PBW and Hemolung^®^), in terms of lung mechanics and gas exchange. We collected arterial blood gases, respiratory and hemodynamic variables, and mixed expired gases at baseline and after 60 minutes of stabilization at *ultraprotective* ventilation. Statistical analysis: 2-tailed Student's t-test. Statistical significance p < 0.05.

## Results

Five men and four women with ARDS where studied (8 pneumonias and 1 abdominal sepsis). Age was 61 ± 14 years, SAPS II at admission 48 ± 28 and ICU mortality 22% (2/9). Seven of these patients were treated with prone positioning during mechanical ventilation. Cannulation was done via femoral vein in all patients, using “ad hoc” 15.5 Fr catheters. Hemolung^®^ allowed a CO_2_ removal rate of 84 ± 9 mL/min, with blood flow 447 ± 35 mL/min, at constant sweep gas flow (10 L/min of O_2_) and pump speed (1400 RPM). Unfractionated heparin dose was 200 ± 78 mg/day and aPTT was 1,56 ± 0.18. During catheter insertion a bolus of 0.6 ± 0.2 mg/kg mg was administered. Hemolung^®^ total days were 5.3 ± 6.2 (range 1 to 22). No significant haemorrhage or hemolysis needing transfusion, device malfunction, insertion and/or withdrawal complications occurred. We report a significant reduction in minute ventilation and alveolar minute ventilation (75% and 66%, respectively), dead space (68%), and driving pressure (69%), without significant changes in arterial blood gases when *ultraprotective* strategy was implemented, as compared to baseline (see tables 1 and 2).

**Table 1:** Table 1

VARIABLE	BASELINE	4ml/kg PBW + Hemolung®	T-TEST p
Vt (mL/kg PBW)	6.4 ± 1	4 ± 0	< 0.001
Vt (mL)	374 ± 55	238 ± 47	< 0.001
RR (bpm)	24 ± 3	28 ± 6	0.027
VE (ml/min) [=Vt*RR]	8798 ± 1297	6639 ± 1679	0.004
PEEP (cmH2O)	11 ± 1	13 ± 4	0.227
Pplat (cmH2O)	24 ± 4	22 ± 3	0.074
Crs (mL/cmH2O) [=Vt/Pplat-PEEP]	30 ± 9	30 ± 11	0.998
ΔP [cmH2O] [=Pplat-PEEP]	13 ± 3	9 ± 6	0.003

**Table 2:** Table 2

VARIABLE	BASELINE	4ml/kg PBW + Hemolung®	T-TEST p
Vd (mL) [=(PaCO2-PECO2/PaCO2]	262 ± 29	175 ± 27	< 0.001
Vd/Vt	0.71 ± 0.06	0.75 ± 0.09	0.219
Va min (mL/min) [=(Vt-Vd)*RR]	2614 ± 771	1718 ± 856	0.028
FiO2	0.6 ± 0.2	0.6 ± 0.1	0.420
pH	7.38 ± 0.06	7.35 ± 0.11	0.493
PaO2 (mmHg)	91 ± 21	109 ± 28	0.138
PaCO2 (mmHg)	50 ± 19	49 ± 12	0.919
MAP (mmHg)	79 ± 18	75 ± 14	0.332
HR (bpm)	101 ± 26	92 ± 22	0.048

## Conclusions

Hemolung^®^ system allows *ultraprotective* ventilation, while maintaining adequate arterial blood gases and significantly decreasing the intensity of ventilator assistance. The technique appears to be useful and safe.

## Grant Acknowledgment

Material for the study was kindly provided by ALung, Pittsburgh, USA.
